# In-hospital survival of critically ill COVID-19 patients treated with glucocorticoids: a multicenter real-world data study

**DOI:** 10.1038/s41598-024-62302-w

**Published:** 2024-05-27

**Authors:** Stefan Angermair, Jan-Hendrik Hardenberg, Kerstin Rubarth, Felix Balzer, Nilufar Akbari, Mario Menk, Claudia Spies, Kai-Uwe Eckardt, Denis Poddubnyy, Britta Siegmund, Thomas Schneider, Sascha Treskatsch

**Affiliations:** 1 Department of Anesthesiology and Intensive Care Medicine, Charité – Universitätsmedizin Berlin, Corporate Member of Freie Universität Berlin and Humboldt-Universität Zu Berlin, Campus Benjamin Franklin, Berlin, Germany; 2 Department of Nephrology and Medical Intensive Care, Charité-Universitätsmedizin Berlin, Corporate Member of Freie Universität Berlin and Humboldt-Universität Zu Berlin, Berlin, Germany; 3 Institute of Medical Informatics, Charité - Universitätsmedizin Berlin, Corporate Member of Freie Universität Berlin and Humboldt-Universität Zu Berlin, Berlin, Germany; 4 Institute of Biometry and Clinical Epidemiology, Charité – Universitätsmedizin Berlin, Corporate Member of Freie Universität Berlin and Humboldt-Universität Zu Berlin, Charitéplatz 1, 10117 Berlin, Germany; 5 Medizinische Fakultät Carl Gustav Carus, Dresden, Germany; 6 Department of Anesthesiology and Intensive Care Medicine, Charité – Universitätsmedizin Berlin, Corporate Member of Freie Universität Berlin and Humboldt Universität Zu Berlin, Campus Virchow-Klinikum and Charité Campus Mitte, Berlin, Germany; 7 Division of Gastroenterology, Infectious Diseases, Rheumatology, Charité - Universitätsmedizin, Corporate Member of Freie Universität Berlin and Humboldt-Universität Zu Berlin, Campus Benjamin Franklin, 10117 Berlin, Germany

**Keywords:** COVID-19, SARS-CoV2, High-dose glucocorticoids, Critical care, Overall survival, Mortality, Critically ill patients, Intensive care unit, Health services, Diseases, Health care

## Abstract

The COVID-19 pandemic has posed a major challenge to healthcare systems globally. Millions of people have been infected, and millions of deaths have been reported worldwide. Glucocorticoids have attracted worldwide attention for their potential efficacy in the treatment of COVID-19. Various glucocorticoids with different dosages and treatment durations have been studied in patients with different severities, with a suitable dosage and treatment duration not yet defined. This study aimed to investigate whether in-hospital survival differs between critically ill patients treated with low-dose glucocorticoids, high-dose glucocorticoids or no glucocorticoids. All critically ill patients admitted to the intensive care unit of the Charité Hospital—Universitätsmedizin Berlin between February 2020 and December 2021 with COVID-19 pneumonia receiving supplemental oxygen were eligible to participate in this multicenter real-world data study. Patients were retrospectively assigned to one of three groups: the high corticosteroid dose (HighC) group (receiving 6 mg parenteral dexamethasone or an equivalent corticosteroid dosage for ten days), the low corticosteroid dose (LowC) group (receiving less than 6 mg parenteral dexamethasone or an equivalent corticosteroid dosage for ten days), or the no corticosteroid (NoC) group. Overall survival and risk effects were compared among groups within the total observation period, as well as at 35 days after the onset of COVID-19 symptoms. Adjusted multivariable Cox proportional hazard regression analysis was performed to compare the risk of death between the treatment groups. Out of 1561 critically ill COVID-19 patients, 1014 were included in the baseline analysis. In the survival study, 1009 patients were assigned to the NoC (n = 346), HighC (n = 552), or LowC group (n = 111). The baseline characteristics were balanced between groups, except for age, BMI, APACHE II score, SOFA and SAPS II. While the 35-day survival did not show any differences, a landmark analysis of the patients surviving beyond 35 days revealed differences between groups. The restricted mean survival time was 112 days in the LowC group [95% CI: 97 – 128], 133 days in the HighC group [95% CI: 124 – 141] and 144 days in the NoC group [95% CI: 121 – 167]. The multivariable-adjusted Cox proportional hazard analysis indicated that, regardless of age, sex, health status or invasive oxygenation, a low-dose treatment increased the hazard of death of critically ill COVID-19 patients by a factor of 2.09 ([95% CI: 0.99, 4.4], *p* = 0.05) and a high-dose corticosteroid treatment increased the risk by a factor of 1.07 ([95% CI: 0.53, 2.15], *p* = 0.85) compared to no treatment with glucocorticoids. The analysis reveals that corticosteroid treatment does not influence the survival of critically ill COVID-19 patients in the intensive care unit within 35 days. Our evaluations further suggest that regardless of ventilation status, the decision-making process for administering corticosteroid therapy should account for the individual severity of the illness.

## Introduction

The COVID-19 pandemic has presented a global health crisis, affecting millions of individuals worldwide. Mortality has been a critical concern, particularly among hospitalized and critically ill COVID-19 patients. Years after the onset of the pandemic, effectively treating patients with COVID-19 remains a challenge^1^. Survival analyses can offer valuable insights into the efficacy of treatment protocols and clinical management strategies for COVID-19 patients. Understanding the factors associated with mortality in this patient population can improve clinical decision-making, resource allocation, and the development of public health policies to improve outcomes for COVID-19 patients.

Throughout the COVID-19 pandemic, glucocorticoids have been widely used as a treatment option for patients with severe COVID-19 illness, especially for those requiring hospitalization and supplemental oxygen^2^. Systemic corticosteroid treatment, as an inexpensive and readily available therapy, has demonstrated positive effects on the disease in patients requiring respiratory support, as shown in randomized controlled trials and high-quality systematic reviews and meta-analyses^3,4^. Glucocorticoids have been proven to reduce mortality in patients with acute respiratory distress syndrome (ARDS), either with or without COVID-19^5,6^. Corticosteroid hormones play a major role in immune system regulation, stress response, and exhibit anti-inflammatory and immunosuppressive actions. However, they have also been shown to exert adverse effects through unspecific suppression of immune responses against viral, bacterial, or fungal pathogens^5,7–10^. Therefore, an ongoing debate surrounds the question of whether a higher dose of glucocorticoids can be effectively used to treat critically ill COVID-19 patients.

The aim of the present study was thus to comprehensively investigate whether overall survival differs among critically ill COVID-19 patients treated with no, low-dose, or high-dose glucocorticoids and to analyze potential causes of death and associated risk factors. The findings of this study could contribute to ongoing efforts to mitigate the impact of COVID-19 on healthcare systems and provide valuable information for future research and clinical practice.

## Materials and methods

### Study design and patients

A multicenter non-interventional, non-controlled, non-randomized, real-world data study was conducted. The study included patients aged 18 years or older admitted to one of the seven COVID-19 intensive care units (ICU) of Charité—Universitätsmedizin Berlin between February 2020 and December 2021. The treatment plan and Standard Operating Procedure (SOP) were consistent across ICUs. The inclusion criteria mandated patients with confirmed COVID-19 pneumonia receiving supplemental oxygen, determined by PCR testing and indicated by ICD-10 codes U07.1, U07.72, or B34.2. Additionally, available data on the last recorded death or contact were required. Patients without COVID-19 as a primary diagnosis in the ICU were excluded.

### Human ethics and consent to participate

The study was approved by the ethics committee of the Charité—Universitätsmedizin Berlin (EA1/270/21) and registered in the German Clinical Trial Register (DRKS ID: DRKS00031724). Informed consent was obtained from each participant or their legal guardian who was enrolled in the study. The study was conducted by the ethical standards outlined in the 19,645 Declaration of Helsinki and its later amendments, or comparable ethical standards.

### Data collection

To gather patients´ information for COVID-19 cases, Structured Query Language (SQL) inquiries were executed using the hospital’s electronic medical record system (COPRA System GmbH, Sasbachwalden, Germany, and SAP AG, Walldorf, Germany). Additionally, COVID-19-related data (PCR results, SARS-CoV2 variant, diagnosis) were retrieved for the respective cases. The physician´s letter also provided details on the primary and secondary diagnoses as well as the duration of the patient´s hospital stay.

### Classification of groups

Included patients were assigned to one of the three groups:

- HighC—patients who received daily parenteral glucocorticoids with 6 mg dexamethasone or 32 mg methylprednisolone or 160 mg hydrocortisone or 40 mg prednisolone for 10 days.
- LowC—patients who received daily doses of less than 6 mg dexamethasone or 32 mg methylprednisolone or 160 mg hydrocortisone or 40 mg prednisolone for 10 days.
- NoC—patients who did not receive any glucocorticoids.

Patients who were taking glucocorticoids as part of their home medication and those receiving low-dose glucocorticoids for a short duration (less than 10 days) were excluded from the study. Similarly, the study did not include patients undergoing treatment with Interleukin-1 or Interleukin-6 inhibitors or antiviral therapies.

### Research questions

The primary aim of the study was to assess differences in overall survival among critically ill COVID-19 patients based on their treatment with no, low-dose, or high-dose glucocorticoids. Patients´ survival was calculated from the index date until their last recorded event, which was either the date of death or the date of discharge from the hospital.

### Statistical analysis

The start date for survival analysis was the first date of COVID-19 symptoms (index date), either before hospital admission or during hospitalization. If not available, the date of the SARS-CoV-2 PCR was used. Patients´ survival was calculated from the index date until the patient’s last recorded event, which was either the date of death or the date of discharge from the hospital. One year was considered to last 365.25 days, and a month was defined as 365.25/12 days.

Kaplan–Meier curves were calculated for all three groups to investigate censoring and proportional hazards (PH) for the primary endpoint “overall survival” and for the competing risk “discharge from hospital”. Additionally, Schoenfeld residuals were investigated. Landmark analyses were performed, splitting the follow-up time at a time point of 35 days. To assess the target estimand, we calculated multivariable Cox proportional hazards models adjusting for the following potential confounders: sex (male/female), invasive oxygen treatment (yes/no), APACHE II score (continuous variable) modelled with b-splines with 3 df, SAPS II score (continuous variable), SOFA score (continuous variable), age (years), urea (continuous variable) modelled with b-splines with 3 df and creatinine (continuous variable). The selection of variables was based on expert background knowledge. Missing values for the APACHE II score were imputed with stochastic regression. In sensitivity analysis, the APACHE II score was excluded to examine the robustness of the results. From the fitted Cox models, we computed the adjusted survival curves as well as adjusted restricted mean survival times (RMST) comparing the three groups. Effect size estimates were reported with 95% confidence intervals (CI) based on the 2.5th and 97.5th percentiles from the distribution of 1000 bootstrap resamples of all patients. Assumptions regarding the Cox model and the causal inference methodology, as well as model-building criteria e.g. the events per variable (EPV) ratio are discussed in the supplement. Continuous variables were described as median with interquartile range (IQR); categorical variables were summarized as absolute and relative frequencies. Data distributions were inspected graphically using box plots and histograms. For all three groups, baseline characteristics and treatment regimens were compared descriptively. All analyses are explorative. Data preparation and analyses were conducted using R statistical software, version 4.1.3 (R Foundation for Statistical Computing). Additional information regarding data distributions, missing data, details on the analysis steps, and sensitivity analysis, can be found in the supplement.

## Results

### Patients

**Figure 1:**
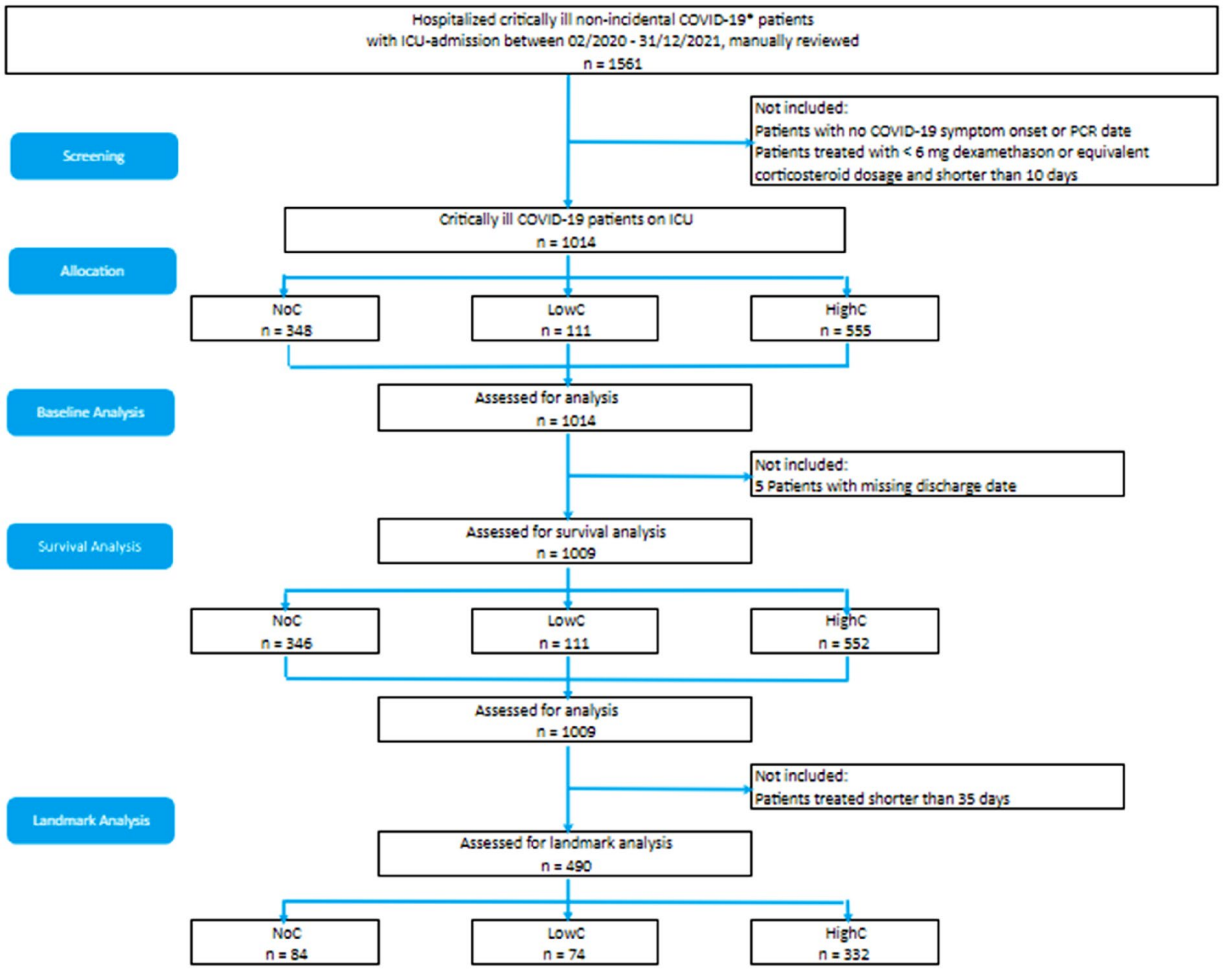
Flow chart of the study population. *Patients had either a positive SARS-CoV-2 PCR test or an ICD-10 code U07.1, U07.72 or B34.2.

Between February 2020 and December 2021, our ICUs admitted 1561 critically ill patients diagnosed with pneumonia, all of whom required supplemental oxygen and were primarily diagnosed with COVID-19. (Fig. 1). Out of these, 547 were excluded from the subsequent analysis. Among the excluded patients, 139 had missing data regarding the onset of COVID-19 symptoms or SARS-CoV-2 PCR results. Additionally, 408 patients received a low dosage of corticosteroid for a short duration (< 10 days). Among the remaining 1009 critically ill COVID-19 patients, 346 patients (34.3%) were treated without glucocorticoids (NoC), 111 patients (11%) received low-dose glucocorticoids for 10 days (LowC), and 552 patients (54.7%) received high-dose glucocorticoids for 10 days (HighC). Patients were followed up for a total of 768 days, with an average follow-up of 45.7 days. Of the 1014 patients, 1009 (99.5%) were eligible for subsequent survival analyses, while five patients were not included due to missing discharge date information. For the landmark analysis, 490 patients who were treated for more than 35 days were included. In the NoC group, 84 patients were examined, in the LowC group 74, and in the HighC group 332 (see Fig. 1).

Table 1 shows the main characteristics of the analyzed patients at baseline. No relevant differences were seen between groups regarding sex, smoking habits, date of COVID-19 onset, comorbidities, Charlson comorbidity index or previous SARS-CoV-2 infections. The sex ratio (male/female) was 2.3 and the median age was 62 years (IQR 53–70) of the total cohort. APACHE II, SOFA and SAPS II scores differed between the groups, with the LowC group having the highest score and the NoC group having the lowest score, with the latter group including patients in a less ill state. Body mass Index was slightly higher in the HighC group than in the other two groups. The median index dates of onset for COVID-19 symptoms between the groups differed slightly with the earliest index date in the NoC and the latest index date in the HighC group. In 1008 of the 1014 patients (99.5%), SARS-CoV-2 infection was their first encounter with the virus.

**Table 1:** Characteristics of critically ill patients with COVID-19 at baseline.

	All patients (N = 1014)	NoC (N = 348)	LowC (N = 111)	HighC (N = 555)
Age at diagnosis, median (IQR), years	62 (53–70)	61 (49–72)	63 (56–68)	62 (54–69)
Sex				
Female, n (%)	304 (30)	100 (28.7)	33 (29.7)	171 (30.8)
Male, n (%)	709 (69.9)	247 (71)	78 (70.3)	384 (69.2)
NA, n (%)	1 (0.1)	1 (0.3)	0	0
Body mass index, mean (min–max), kg/m^*2*^	29 (26–35)	28 (26–34)	29 (26–35)	29 (26–35)
Smoker				
Past, n (%)	110 (10.8)	34 (9.8)	13 (11.7)	63 (11.4)
Active, n (%)	58 (5.7)	18 (5.2)	4 (3.6)	36 (6.5)
NA, n (%)	846 (83.4)	296 (85.1)	94 (84.7)	456 (82.2)
Comorbidities, n (%)				
Hypertension, n (%)	466 (46)	173 (49.7)	50 (45)	243 (43.8)
Asthma, n (%)	72 (7.1)	22 (6.3)	6 (5.4)	44 (7.9)
Diabetes Type 1, n (%)	9 (0.8)	3 (0.9)	3 (2.7)	3 (0.5)
Diabetes Type 2, n (%)	260 (25.6)	83 (23.9)	28 (25.2)	149 (26.8)
Coronary artery disease, n (%)	148 (14.6)	55 (15.8)	14 (12.6)	79 (14.2)
Fibrosis Lung, n (%)	9 (1.0)	3 (0.9)	0	6 (1.1)
HIV, n (%)	6 (0.6)	4 (1.2)	0	2 (0.4)
Depression, n (%)	46 (4.5)	12 (3.4)	7 (6.3)	27 (4.9)
Dementia, n (%)	39 (3.8)	19 (5.5)	2 (1.8)	18 (3.2)
Malignoma, n (%)	47 (4.6)	16 (4.6)	6 (5.4)	25 (4.5)
Kidney transplantation, n (%)	28 (2.8)	6 (1.7)	2 (1.8)	20 (3.6)
NA, n (%)	4 (0.4)	1 (0.3)	0	3 (0.5)
SARS-CoV-2 1^*st*^* infection, n (%)*	1008 (99.4)	348 (100)	109 (98.2)	551 (99.3)
SARS-CoV-2 (Feb 2020 – Oct 2020), n (%)	169 (16.7)	90 (25.9)	14 (12.6)	65 (11.7)
Glucocorticoids, n (%)	79 (46.7)	90 (53.3)	14 (8.3)	65 (38.4)
SARS-CoV-2 (Oct 2020 – Dec 2021), n (%)	845 (83.3)	258 (74.1)	97 (87.4)	490 (88.3)
Glucocorticoids, n (%)	587 (69.5)	258 (30.5)	97 (11.5)	490 (58)
APACHE II score, median (IQR)	23 (15–30)	16 (11–24)	29 (20–35)	25 (17–31)
SOFA score, median (IQR)	7 (2–11)	2.5 (0–5.8)	10 (6.5–14)	8 (4–12)
SAPS II score, median (IQR)	38 (30–46)	32 (25–41)	43 (35–51)	39 (31–48)
Charlson comorbidity index, median (IQR)	2 (1–4)	2 (1–4)	2 (1–4)	2 (1–4)
*Creatinine, median (IQR)*	0.93 (0.67–1.5)	0.85 (0.69–1.2)	1.4 (0.73–2.2)	0.99 (0.66–1.6)
*Urea, median (IQR)*	59 (37–90)	45 (30–68)	73 (50–100)	64 (43–100)
*Renal replacement therapy, n (%)*	434 (42.9)	45 (12.9)	84 (75.7)	305 (55)
Invasive Ventilation, n (%)	710 (70)	120 (34.5)	100 (90.1)	490 (88.3)
Norepinephrine, n (%)	732 (72.2)	130 (37.4)	100 (90.1)	502 (90.5)

Percentages of sub-characteristics may not sum up to 100% due to rounding procedures. APACHE II, Acute physiology and chronic health evaluation; Feb, February; HighC, High corticosteroid dose; HIV, Human immunodeficiency virus; IQR, Interquartile range; LowC, Low corticosteroid dose; n, Number; NA, Not available; NoC, No corticosteroid dose; Oct, October; SAPS II, Simplified acute physiology score; SOFA, Sepsis-related organ failure assessment score; Dec, December.

#### 35-day survival

**Figure 2:**
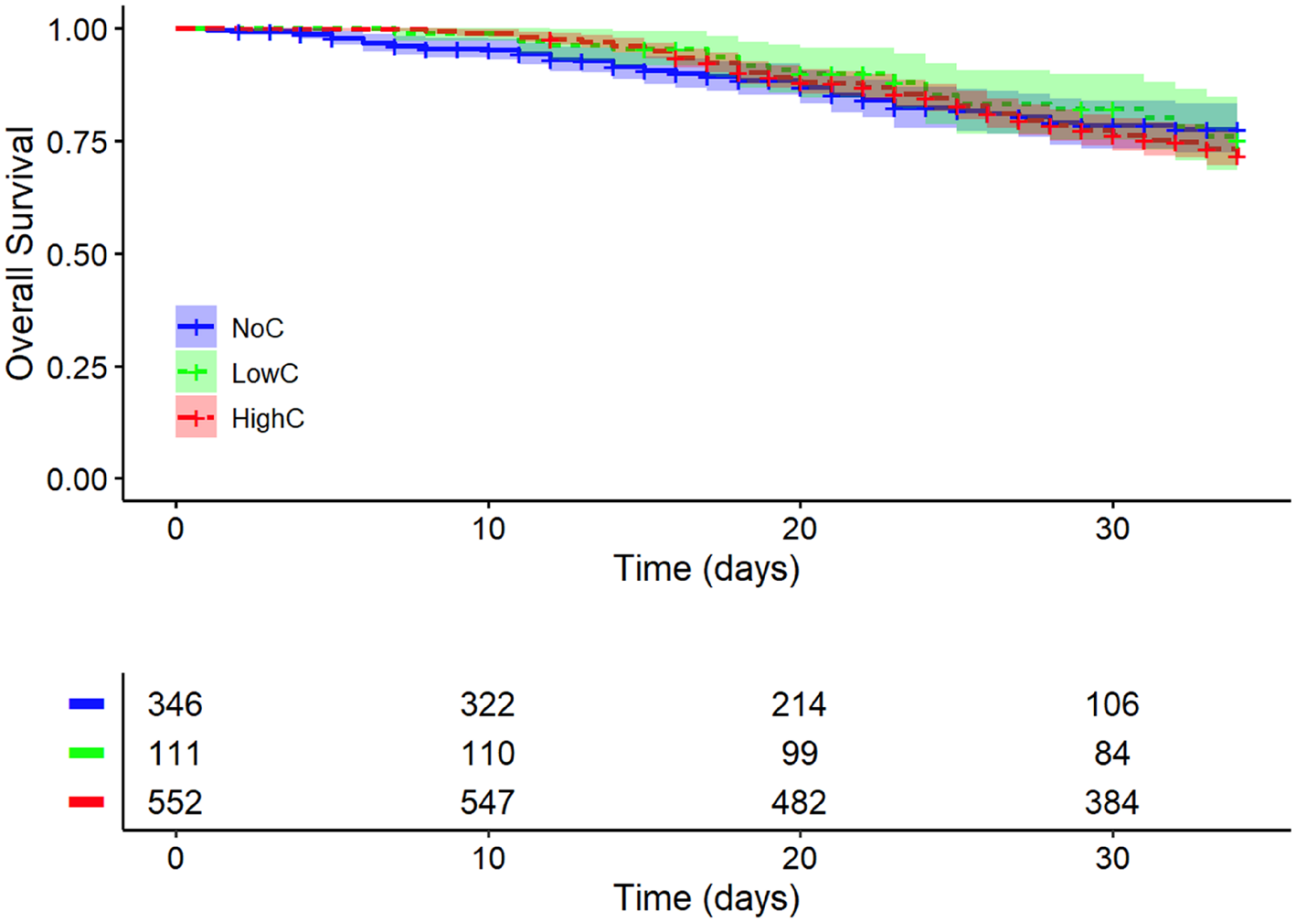
35-Day in-hospital survival. Kaplan–Meier survival curves showing overall survival of COVID-19 patients treated with either NoC (blue line), LowC (green line) or HighC (red line), n = 1009; NoC, No corticosteroid dose; LowC, Low corticosteroid dose; HighC, High corticosteroid dose.

There was no relevant difference between the three groups in the 35-day survival analysis. The 35-day survival rates were 77.5% [95% CI: 72.2, 83.1] for patients in the NoC group, 75.2% [95% CI: 67.3, 84.0] for patients in the LowC and 71.7% [95% CI: 67.9, 75.7] for patients in the HighC group (see Fig. 2).

### In-hospital overall survival

**Figure 3:**
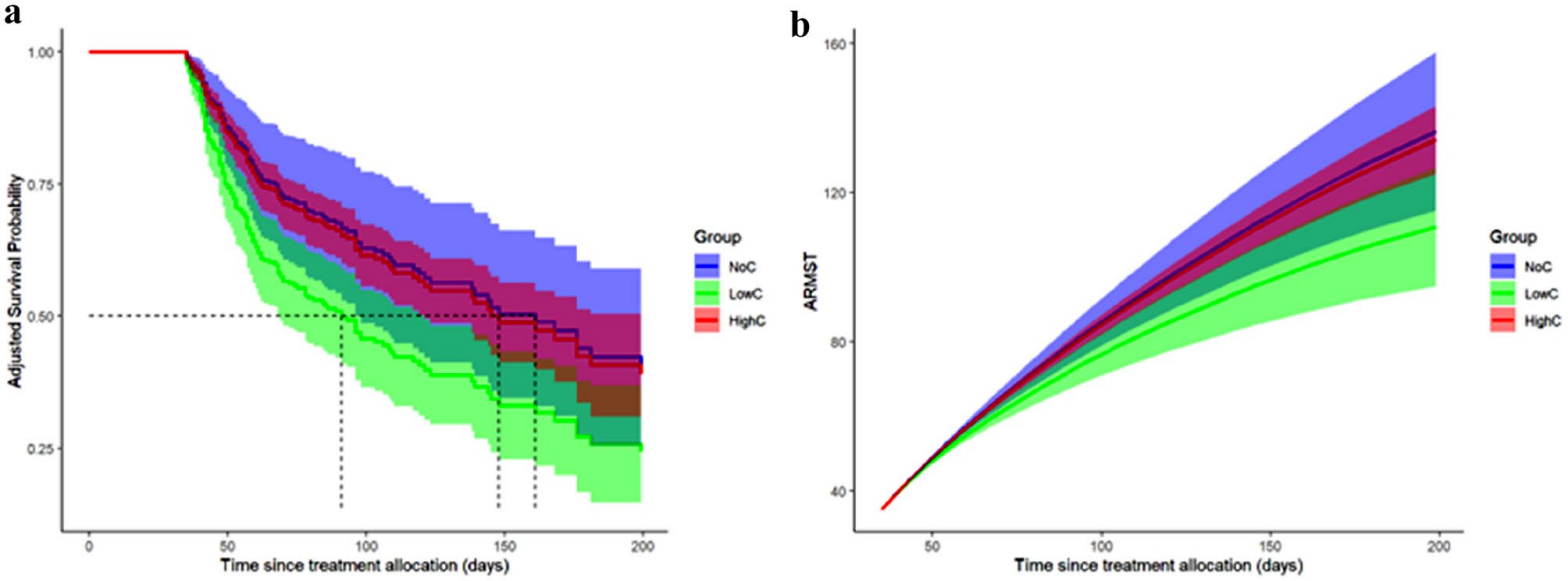
Overall survival. Adjusted survival curves (3a) for the landmarking time point of 35 days showing the overall survival of COVID-19 patients treated with either NoC (blue line), LowC (green line) or HighC (red line); n = 490 (**a**) Adjusted Survival Probability in the NoC with 144 days [95% CI: 121, 167], LowC 112 days [95% CI 97, 128] and HighC 133 days [95% CI: 124, 141] (**b**) Adjusted restricted mean survival time. NoC, no corticosteroid dose; LowC, low corticosteroid dose; HighC, high corticosteroid dose; ARMST, adjusted restricted mean survival time.

490 critically ill COVID-19 patients were included in the landmark analyses after 35 days with 84 patients (17%) in the NoC group, 74 patients (15%) in the LowC group and 332 patients (68%) in the HighC group (see Fig. 3). The NoC group exhibited a restricted mean survival of 144 days [95% CI: 121, 167], surpassing both the HighC group with a restricted mean survival of 133 days [95% CI: 124, 141] and the LowC group with a restricted mean survival of 112 days [95% CI 97, 128] (see Fig. 3a).

### Length of stay (LOS)

**Figure 4:**
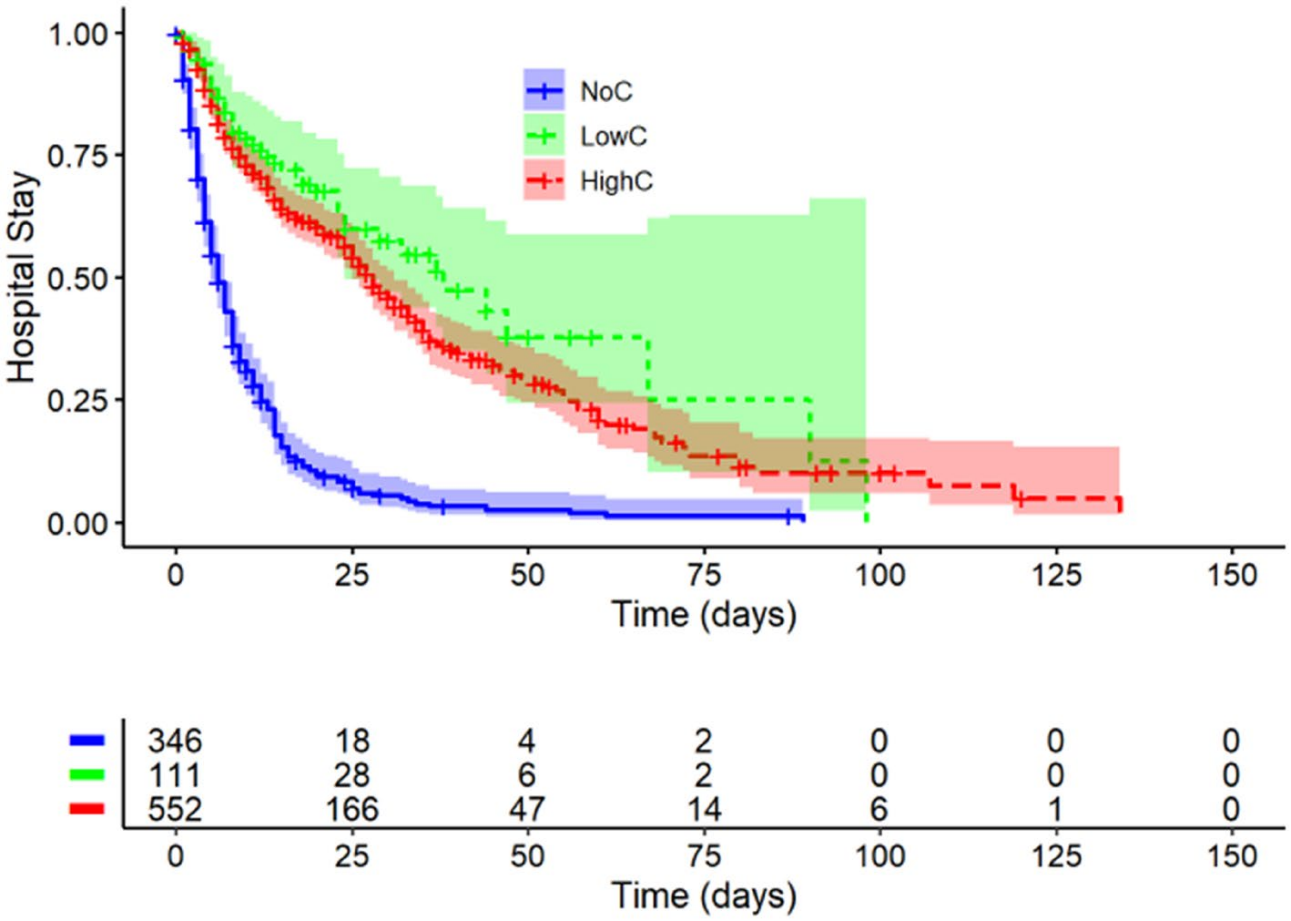
Length of stay. Kaplan–Meier curve displays the time of patients without glucocorticoids (NoC), with high-dose glucocorticoids (HighC) and low-dose glucocorticoids (LowC) in the ICU. NoC (blue line), LowC (green line) or HighC (red line), n = 1009; NoC, No corticosteroid dose; LowC, Low corticosteroid dose; HighC, High corticosteroid dose.

1009 patients were assessed upon discharge from the hospital, with 346 (39%) patients in the NoC, 552 patients (55%) in the HighC and 111 patients (11%) in the LowC group (see Fig. 4). All patients were discharged alive from the ICU.

### Cox proportional hazard analysis

Adjusted multivariable Cox proportional hazard analyses were conducted to estimate the impact of corticosteroid treatment on ICU mortality. The analysis was adjusted for the variables sex, invasive oxygen, APACHE II, SAPS II, SOFA, age, creatinine and urea, with a sample size of 490 and 168 events (EPV = 12). It was found that high-dose treatment increased the adjusted hazard of dying by a factor of 1.07 ([95% CI: 0.53, 2.15], *p* = 0.85), while low-dose glucocorticoids showed an increase by a factor of 2.1 ([95% CI: 0.99, 4.4], *p* = 0.05), compared to no corticosteroid treatment. In sensitivity analysis, we excluded the APACHE II score, due to the high number of missing values that needed to be imputed to examine the robustness of the results. The results remained almost unchanged (see supplement).

## Discussion

In this study, we investigated the influence of different corticosteroid doses on the overall survival of critically ill COVID-19 patients with pneumonia who require oxygen supplementation. Our real-world data study results suggest that patients who are not severely critically ill in the intensive care unit may not necessarily benefit from corticosteroid therapy.

Glucocorticoids act as potent immune modulators that can prevent or attenuate hyper-inflammatory states typical of severe SARS-CoV-2 infections through genomic and/or non-genomic effects^11^. Glucocorticoids, such as dexamethasone, have shown promising results in reducing mortality rates among COVID-19 patients^3–5,11,12^. A meta-analysis demonstrated that the use of glucocorticoids in critically ill COVID-19 patients significantly reduced the 28-day mortality^2^. International guidelines universally recommend the use of glucocorticoids for hospitalized COVID-19 patients with acute respiratory failure, supported by moderate-to-high-quality evidence^2,13^. A systematic review and meta-analysis substantiate the notion that glucocorticoids may decrease mortality and duration of mechanical ventilation in patients with ARDS, including those with COVID-19^6^. Various glucocorticoids with different doses and durations have been studied in patients with different severities^14^. However, the appropriate dose and duration of corticosteroid therapy for patients with COVID-19 have not been clearly defined. After the RECOVERY study, it is recommended to use dexamethasone at a dosage of 6 mg once daily for a duration spanning 7 to 10 days^4^. In the RECOVERY study, patients who received 6 mg/d of dexamethasone had a decreased mortality. The difference in all patients' mortality was 22.9% in those who received dexamethasone and 25.7% in those who did not^4^. However, Covello et al. reported in a 2023 meta-analysis that the use of glucocorticoids is likely to increase mortality in hospitalized COVID-19 patients who are not receiving oxygen^15^. The author concludes that administering glucocorticoids to patients without supplemental oxygen may be harmful. In our study, the patient population is highly heterogeneous, with a wide range of pre-existing conditions and varying disease severity upon ICU admission but all patients required supplemental oxygen upon admission to the intensive care unit due to respiratory insufficiency. Other clinical trials have observed that treatment with dexamethasone in patients with respiratory insufficiency associated with moderate ARDS and COVID-19 had no significant effect on all-cause mortality, ICU-free days or duration of mechanical ventilation at 28 days^16,17^. Glucocorticoids appear to elicit distinct outcomes in patients with COVID-19^18^. Covello et al. speculate that in patients sick enough to receive oxygen treatment, the anti-inflammatory effects of glucocorticoids, as opposed to the effects on delayed viral clearance, lead to selective positive effects^15^.

Our real-world analysis of the COVID-19 cohort has now confirmed these findings demonstrating no relevant difference in 35-day in-hospital survival rates among patient groups treated without glucocorticoids, with low-dose glucocorticoids or with high-dose glucocorticoids for 10 days. Additionally, our study analyzed the survival rates of critically ill patients up to 200 days. Interestingly, we observed no relevant increase in mortality among critically ill COVID-19 patients receiving corticosteroid treatment with doses of 6 mg/d of dexamethasone, compared to patients without glucocorticoid treatment, and a 2.09-fold increase for lower doses. This effect was measured while adjusting for age, sex, invasive oxygenation or disease status upon admission such as APACHE 2, SAPS II or SOFA. Patients without corticosteroid therapy upon admission to the ICU had the lowest scores on APACHE 2, SOFA and SAPS II. The results also aligned with the analysis indicating that patients in the NoC group had the shortest duration of stay in hospital, while the LowC group stayed the longest.

Our finding thus aligns with existing literature, suggesting that corticosteroid treatment in different doses may not yield benefits for all critically ill COVID-19 patients. Many recently published studies focused on the question of the effectiveness of different dosages of dexamethasone in COVID-19 patients compared to the standard dosage. The COVID STEROID 2 trial revealed no discernible difference in days survived without life support when comparing dexamethasone at 12 mg versus 6 mg^19^. Taboada et al. observed a decrease in clinical deterioration within 11 days using dexamethasone at 20 mg as opposed to the standard 6 mg dose, though without any improvement in 28-day mortality^20^. Additionally, trials by Maskin and Wu failed to demonstrate any advantages of higher dexamethasone doses concerning clinically relevant endpoints^17^. Notably, another randomized controlled trial in COVID-19 patients receiving high-flow oxygen or non-invasive ventilation found that a higher dose of dexamethasone (20 mg daily) was even associated with increased 28-day mortality^21^.

Furthermore, long-term corticosteroid use has also been associated with higher mortality rates (long-term HR 1.68; [95% CI: 1.16—2.45]) in mechanically ventilated COVID-19 patients with ARDS^22,23^. One potential explanation for the observed adverse effects of corticosteroid treatment might be their unspecific suppression of various immune response pathways against pathogens^9,10^. Corticosteroid therapy is associated with certain adverse events, including hyperglycemia, hypernatremia, and neuromuscular weakness^3^. Differences in doses can be responsible for diverse clinical efficacy in various settings, as they critically influence the activity of the glucocorticoid receptor^24^.

In our analysis, patients administered a dexamethasone dosage of less than 6 mg for 10 days showed prolonged stays in the ICU and an elevated risk of mortality. Notably, individuals in the LowC group exhibited the highest disease severity upon admission, potentially resulting in a reduced corticosteroid dosage.

Nevertheless, as we systematically considered confounding factors, the observed effect appears to diminish. However, low doses of glucocorticoids gradually activate their effects, while high doses can rapidly saturate cytoplasmic glucocorticoid receptors, leading to a swift onset of anti-inflammatory action^25^. A higher proportion of genomic effects is emphasized when using low-dose steroids, whereas an increasing number of non-genomic effects is typically associated with higher doses^25^. In the literature, conflicting findings regarding the benefits of glucocorticoids can be attributed to various factors, including differences in patient cohorts, duration of corticosteroid treatment, and length of follow-up in mortality assessments conducted in respective clinical studies. The latest guidelines developed by the European Respiratory Society (ERS) and the WHO identified the need to better evaluate the optimal glucocorticoids to be used in COVID-19 in terms of formulation, dosage, timing, and the scheme of administration^13,20^.

Our evaluations suggest that the decision-making process for administering corticosteroid therapy should account for the individual severity of the illness. As our understanding of the disease, progressed subsequent studies were conducted to investigate long-term survival rates and outcomes, aiming to gain a deeper understanding of prognosis and the effectiveness of various treatment approaches^26,27^. Many of these later studies with extended observation periods, however, were observational^26–28^, like the present real-world data (RWD) study, which complemented the findings of randomized controlled trials (RCTs).

## Limitations

Our study has several limitations that should be acknowledged. Firstly, it is important to note that our study design was non-randomized, and despite adjusting for confounding factors in our multivariable Cox regression analyses, there may still be unknown confounders that have influenced our findings. Secondly, adverse events related to both high-dose and low-dose glucocorticoids were not extensively reported. Additionally, the analysis cannot make any statement regarding the severity of pneumonia. Long-term follow-up outcomes for COVID-19 survivors treated with high-dose and low-dose glucocorticoids are essential to assess potential complications, particularly in the context of ARDS and pneumonia relapse. Therefore, further studies with a sufficient follow-up length will need to be necessary to evaluate the effectiveness of different treatment strategies.

## Conclusions

The analysis indicates that corticosteroid treatment does not influence the survival of critically ill COVID-19 patients in the intensive care unit within 35 days. Furthermore, our assessments suggest that irrespective of ventilation status, the individual severity of the illness should be considered in the decision-making process for administering corticosteroid therapy. Our study contributes to the existing knowledge on the clinical impact of corticosteroid use in critically ill COVID-19 patients. It is crucial to emphasize that our research is based on a real-world data study, adding a practical dimension to the presented evidence. Nevertheless, it is essential to interpret the results with caution due to the nature of real-world data in our study.

### Supplementary Information

Supplementary Information.

## Data Availability

The datasets generated during and/or analyzed during the current study are available from the corresponding author upon reasonable request.
