# Supplementary material for: In-hospital survival of critically ill COVID-19 patients treated with glucocorticoids: a multicenter real-world data study
Source: Sci Rep. 2024 May 27;14:12138. doi: 10.1038/s41598-024-62302-w (PMC11130266; doi:10.1038/s41598-024-62302-w)
Supplement: Supplementary file 1 — Supplementary Information. [file 41598_2024_62302_MOESM1_ESM.docx]

# Methods: Data distribution and analysis approach

## Data management and handling of missing data

We analyzed data distributions and missing values. Supp. Fig. 1 illustrates the distributions of age, creatinine, urea, APACHE II, SAPS II, and SOFA score. Creatinine, urea, SOFA score, CRP, PCT, and IL-6 exhibit right-skewed patterns, whereas the remaining variables demonstrate approximately symmetric distributions. Notably, for IL-6, all patients with values exceeding 5000 were capped at 5000 to enhance the graphical representation for lower values.


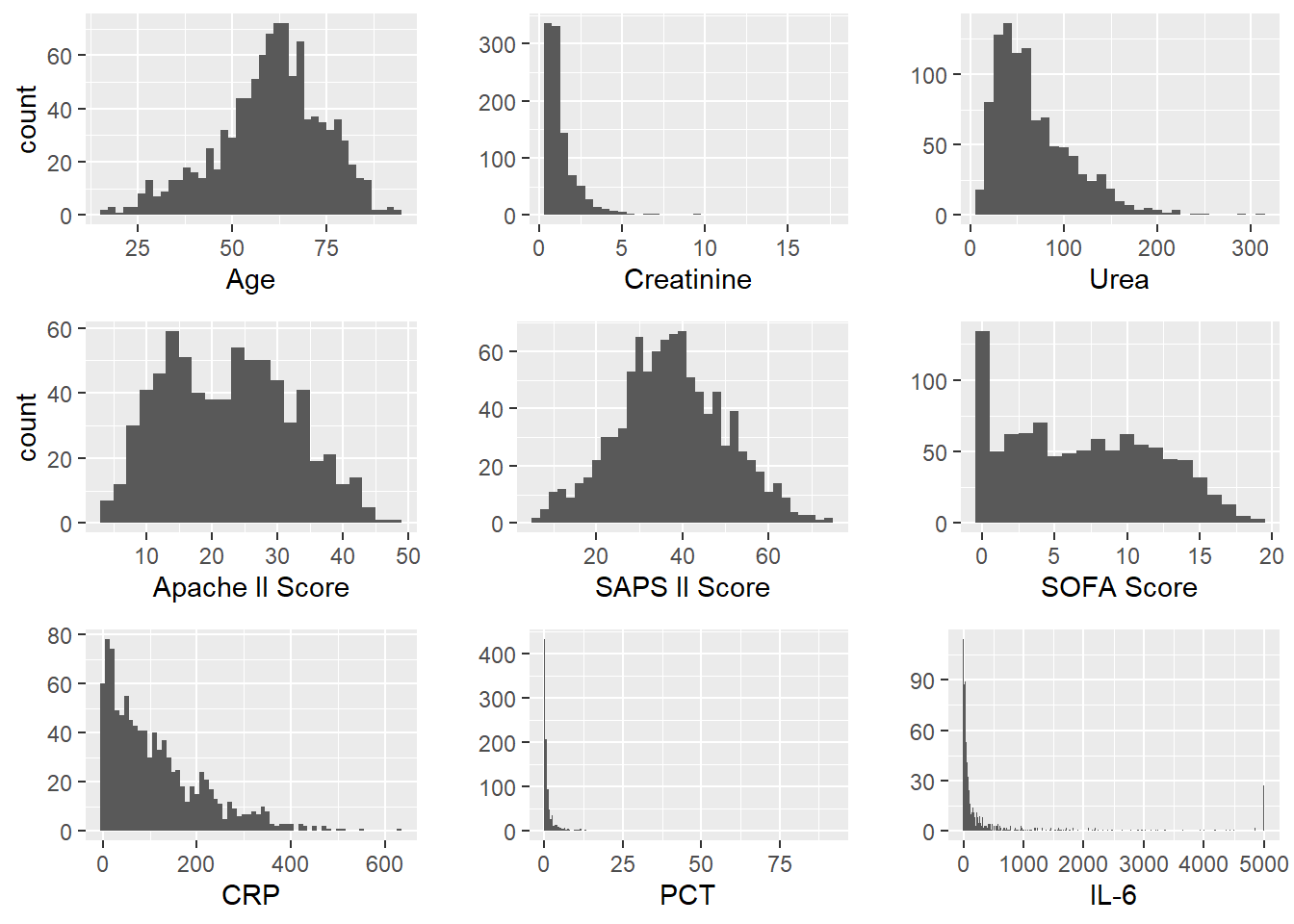


**Supp. Figure. 1: Data distributions**

Supp. Table 1 in the paper overviews the count and percentage of missing values across all variables. The subsequent two figures present the percentage of missing values specifically of the survival endpoint and all potential confounding factors. The left figure encompasses the entire study population, while the right figure focuses on patients who survived up to the landmark time point of 35 days. Given the substantial number of missing values associated with the APACHE II score, we addressed this issue by employing stochastic regression imputation techniques.


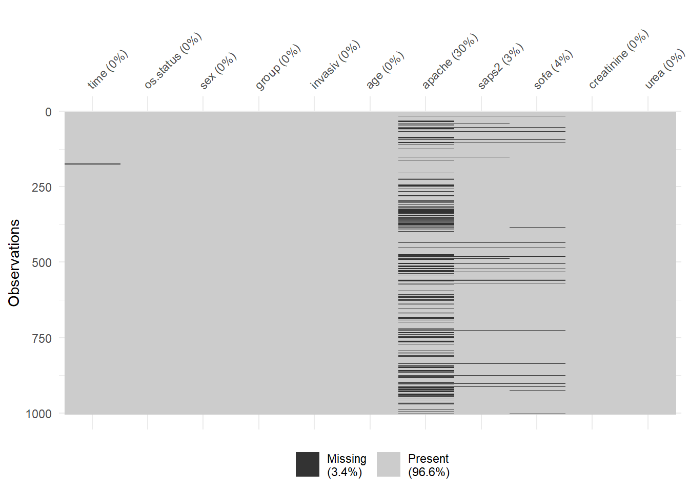

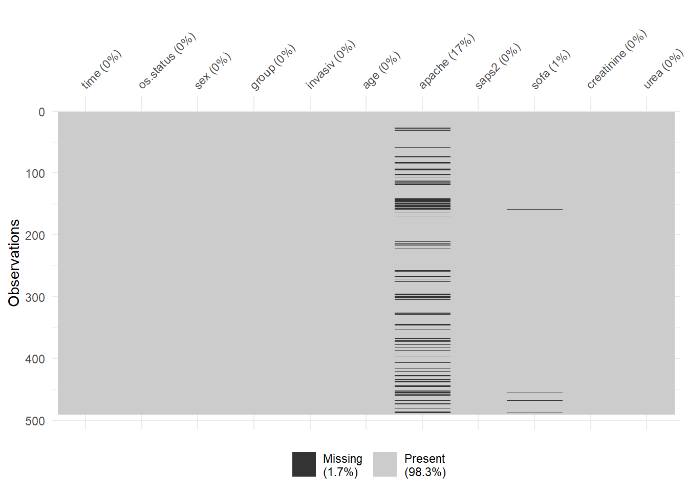


**Supp. Table 1: Missing Data for Apache II scoring:** The left table shows the entire study population and the right table shows patients who survived up to the landmark time point of 35 days.

Supp. Table 2 shows the baseline characteristics for patients who survived until the landmarking time point.

|  | **All patients** **(N=490)** | **NoC** **(N=84)** | **LowC** **(N=74)** | **HighC** **(N=332)** |
| --- | --- | --- | --- | --- |
| **Age at diagnosis, median (IQR), years** | 61 [53-67] | 58 [50-66] | 63 [54-67] | 61 [53-67] |
| **Sex** |  |  |  |  |
| Female, n (%) | 133 (27.1) | 17 (20.2) | 21 (28.4) | 95 (28.6) |
| Male, n (%) | 357 (72.9) | 67 (79.8) | 53 (71.6) | 237 (71.4) |
| **Body mass index, median (IQR), kg/m2** | 29 [26-35] | 29 [26-34] | 29 [26-32] | 29 [26-35] |
| NA, n (%) | 83 (16.9) | 20 (23.8) | 17 (23.0) | 46 (13.9) |
| **Smoker** |  |  |  |  |
| Active, n (%) | 21 (4.3) | 3 (3.6) | 2 (2.7) | 16 (4.8) |
| Past, n (%) | 60 (12.2) | 10 (11.9) | 11 (14.9) | 39 (11.7) |
| NA, n (%) | 409 (83.5) | 71 (84.5) | 61 (82.4) | 277 (83.4) |
| **Date of COVID-19 onset, month/year, median (IQR)** | Jan21 [Nov20- Mai21] | Dez20 [Okt20- Apr21] | Feb21 [Dez20- Sep21] | Feb21 [Nov20-Jun21] |
| **Comorbidities** |  |  |  |  |
| Hypertension, n (%) | 261 (53.3) | 41 (48.8) | 41 (55.4) | 179 (53.9) |
| Asthma, n (%) | 36 (7.3) | 4 (4.8) | 5 (6.8) | 27 (8.1) |
| Diabetes Type 1, n (%) | 2 (0.4) | 0 (0) | 2 (2.7) | 0 (0) |
| Diabetes Type 2, n (%) | 114 (23.3) | 15 (17.9) | 17 (23.0) | 82 (24.7) |
| Coronary artery disease, n (%) | 50 (10.2) | 6 (7.1) | 9 (12.2) | 35 (10.5) |
| Fibrosis Lung, n (%) | 6 (1.2) | 2 (2.4) | 0 (0) | 4 (1.2) |
| HIV, n (%) | 3 (0.6) | 1 (1.2) | 0 (0) | 2 (0.6) |
| Depression, n (%) | 18 (3.7) | 2 (2.4) | 5 (6.8) | 11 (3.3) |
| Dementia, n (%) | 3 (0.6) | 0 (0) | 0 (0) | 3 (0.9) |
| Malignoma, n (%) | 14 (2.9) | 5 (6.0) | 2 (2.7) | 7 (2.1) |
| Kidney transplantation, n (%) | 14 (2.9) | 2 (2.4) | 2 (2.7) | 10 (3.0) |
| NA, n (%) | 1 (0.2) | 1 (1.2) | 0 (0) | 0 (0) |
| **COVID-19 first infection, n (%)** | 489 (99.8) | 84 (100) | 73 (98.6) | 332 (100) |
| **Charlson comorbidity index, median (IQR)** | 2.0 [1.0-3.0] | 2.0 [1.0-3.0] | 2.0 [1.0-4.0] | 2.0 [1.0-3.0] |
| **APACHE II, median [Q1, Q3]** | 25 [17-31] | 22 [14-28] | 30 [21-35] | 25 [17-31] |
| NA, n (%) | 85 (17.3) | 17 (20.2) | 13 (17.6) | 55 (16.6) |
| **SAPS II, median [Q1, Q3]** | 37 [31-44] | 33 [26-40] | 44 [35-51] | 37 [31-44] |
| **SOFA, median [Q1, Q3]** | 8.0 [4.0-11] | 5.0 [1.8-8.0] | 10 [7.0-14] | 8.0 [4.0-11] |
| NA, n (%) | 4 (0.8) | 4 (4.8) | 0 (0) | 0 (0) |
| **Creatinine, median [Q1, Q3]** | 0.89 [0.58-1.5] | 0.77 [0.58-1.1] | 1.3 [0.73-1.9] | 0.87 [0.55-1.4] |
| **Urea, median [Q1, Q3]** | 60 [42-93] | 49 [31-82] | 72 [51-91] | 63 [42-98] |
| **Renal Replacement Therapy, n (%)** | 309 (59.2) | 49 (38.6) | 63 (82.9) | 198 (57.7) |
| **Norepinephrine, n (%)** | 454 (92.7) | 62 (73.8) | 72 (97.3) | 320 (96.4) |
| **Invasive Ventilation, n (%)** | 424 (95.5) | 49 (86.0) | 68 (97.1) | 307 (96.8) |
| **Procalcitonin, median [Q1, Q3]** | 0.45 [0.17-1.7] | 0.29 [0.15-0.94] | 1.1 [0.39-2.4] | 0.45 [0.15-1.6] |
| NA, n (%) | 1 (0.2) | 1 (1.2) | 0 (0) | 0 (0) |
| **CRP, median [Q1, Q3]** | 89 [37-180] | 69 [27-150] | 140 [42-240] | 88 [39-160] |
| NA, n (%) | 2 (0.4) | 0 (0) | 0 (0) | 2 (0.6) |
| **Il-6, median [Q1, Q3]** | 61 [27-190] | 52 [23-110] | 81 [41-240] | 59 [26-210] |
| NA, n (%) | 10 (2.0) | 4 (4.8) | 2 (2.7) | 4 (1.2) |

**Supp. Table 2: Characteristics of critically ill patients with COVID-19 at landmark**. Percentages of sub-characteristics may not sum up to 100% due to rounding procedures. APACHE, Acute Physiology and Chronic Health Evaluation; CRP, c-reactive protein; HighC, high corticosteroid dose; IQR, interquartile range; Il-6, Interleukin 6; LowC, low corticosteroid dose; n, number; NA, not available; NoC, no corticosteroid dose; SAPS, Simplified Acute Physiology Score; SOFA, Sepsis-related organ failure assessment score.

## Assumptions

The applied causal inference methodology relies on several structural assumptions:

• Pretreatment covariates - all confounders were measured before treatment allocation.

• Unmeasured confounding - we cannot guarantee that our information on measured confounders was sufficient to ensure the exchangeability of our treatment groups. Nonetheless, we adjusted for the most relevant confounders to ensure that the Events Per Variable (EPV) remained sufficient.

• Positivity - Each participant had a positive probability of receiving any corticosteroid dosage. There is no indication of a violation of the positivity assumption.

• Consistency for all three groups – In considering consistency, it is essential to note that the dosage groups have ranges. As specified in the group classification, patients were included in the LowC or HighC group if they had taken the medication for 10 days.

• No interference – The assumption of no interference is plausible since the potential outcomes of patients do not depend on the treatment assignment of other patients.

Additionally, the application of the Cox model assumes the proportional hazards assumption. Concerning the entire population, hazards are non-proportional, as evident in the Kaplan-Meier curves. However, our primary focus was after the landmark time point of 35 days, during which we did not observe any significant departures from the proportional hazards assumption.

## Sensitivity Analysis

Subsequently, we will show two different adjustment models. It is important to note that the confounders are not to be interpreted as {Westreich, 2013 #40}risk factors [1]{Westreich, 2013 #40}. The subsequent models only include the most necessary non-linear functional forms, to still keep a minimal EPV ratio of 10. Although this is below the recommended minimal ratio by Harrell of 1:15, we found it more important to include the essential confounders since the aim of this model is explanatory [2].

Adjustment Method 1 – Stochastic Regression Imputation for APACHE II:

The Cox proportional hazards model was employed, adjusting for the following confounders: sex (male/female), age (years), invasive oxygen treatment (yes/no), SAPS 2 score (continuous variable) modelled with B-splines using 3 degrees of freedom (df), SOFA score (continuous variable), urea (continuous variable) modelled with B-splines using 3 df, and creatinine (continuous variable). The sample size comprises 486 subjects, with 168 events observed. The Effective Predictors of Variables (EPV) is 12. The model's summary is presented in the table below, and the subsequent figure displays the flexible modelling of continuous variables.

| Variable | HR | low CI | high CI | Std. error | P value |
| --- | --- | --- | --- | --- | --- |
| LowC | 2.085 | 0.989 | 4.397 | 0.381 | 0.053 |
| HighC | 1.069 | 0.531 | 2.150 | 0.357 | 0.852 |
| Male | 0.994 | 0.670 | 1.476 | 0.202 | 0.977 |
| Age | 1.013 | 0.997 | 1.029 | 0.008 | 0.123 |
| Invasive | 0.390 | 0.135 | 1.122 | 0.540 | 0.081 |
| APACHE II | 1.006 | 0.989 | 1.023 | 0.009 | 0.486 |
| SOFA | 1.178 | 1.127 | 1.230 | 0.022 | 0.000 |
| Creatinine | 0.812 | 0.657 | 1.004 | 0.108 | 0.054 |

**Supp. Table 3: Results of Cox proportional hazards model with confounders.** Reference group: NoC, no corticosteroid dose. APACHE, Acute Physiology and Chronic Health Evaluation; Cl, confidence limit; Error, standard error; HR, hazard ratio; HighC, high corticosteroid dose; Invasive, invasive ventilation; LowC, low corticosteroid dose; SOFA, Acute Physiology and Chronic Health Evaluation.


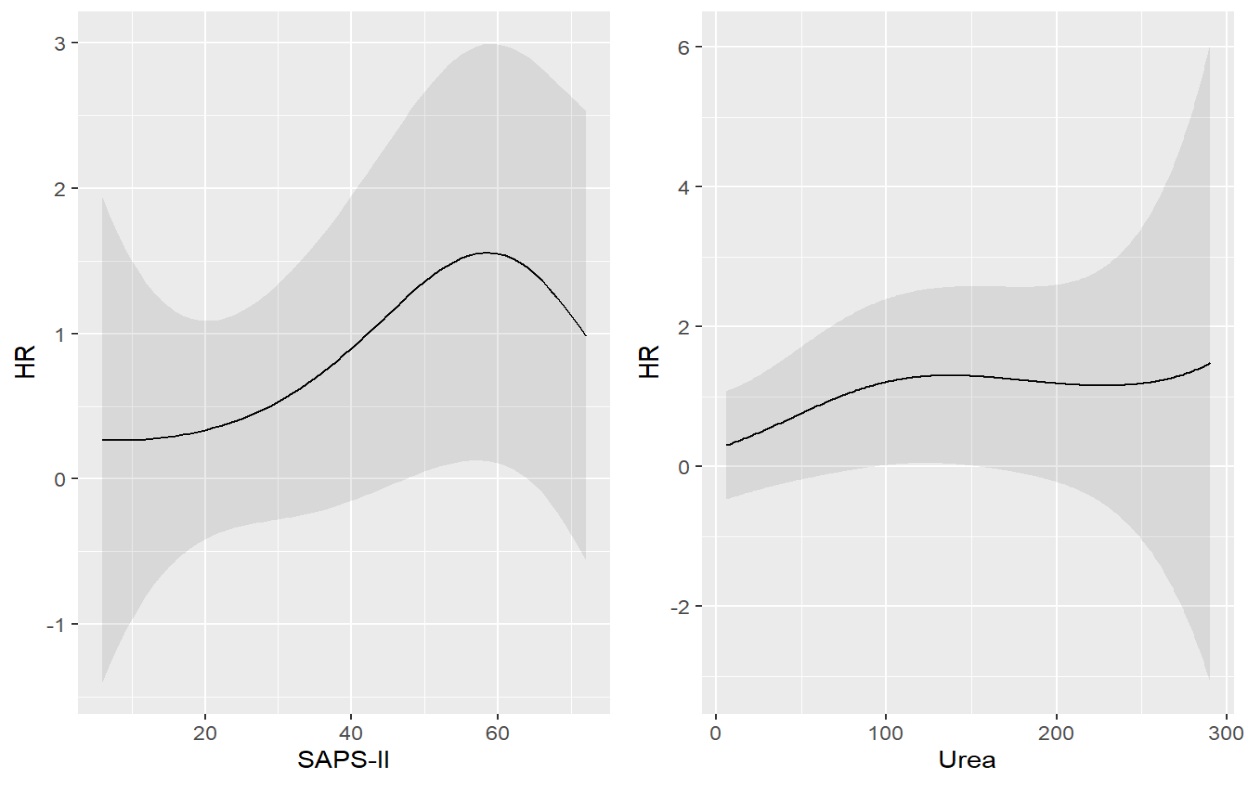


**Supp. Figure 2: Confounders that were modelled with b-splines and 3 degrees of freedom.** SAPS, Simplified Acute Physiology Score.

3a) 3b)


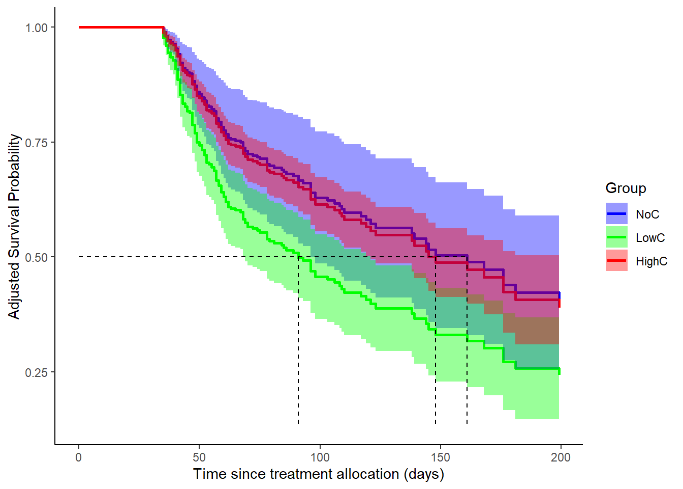

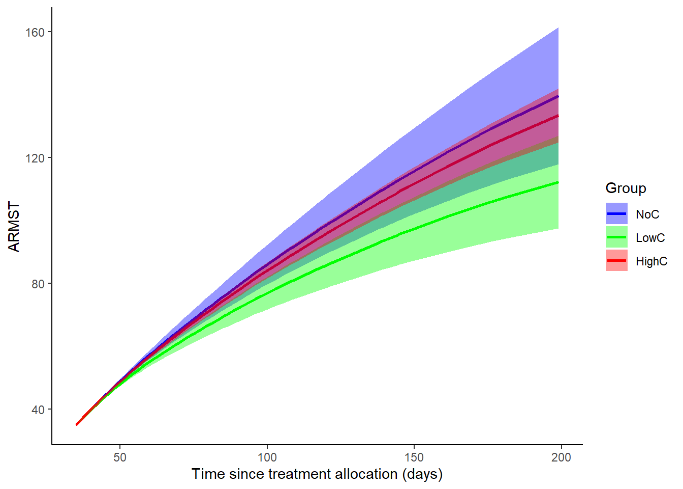
**Supp. Figure 3: Overall survival.** Adjusted survival curves (3a) for the landmarking time point of 35 days showing the overall survival of COVID-19 patients treated with either NoC (blue line), LowC (green line) or HighC (red line); n= 486. **3a.** Adjusted Survival Probability and **3b.** Adjusted restricted mean survival time. NoC, no corticosteroid dose; LowC, low corticosteroid dose; HighC, high corticosteroid dose; ARMST, adjusted restricted mean survival time.

Adjustment Method 2 – excluding APACHE II score:

The Cox proportional hazards model was employed, adjusting for the following confounders: sex (male/female), age (years), invasive oxygen treatment (yes/no), SAPS2 score (continuous variable) modelled with B-splines using 3 degrees of freedom (df), SOFA score (continuous variable), urea (continuous variable) modelled with B-splines using 3 df, and creatinine (continuous variable). The sample size comprises 486 subjects, with 168 events observed. The Effective Predictors of Variables (EPV) is 12.9. The model is summarized in the table below, and the subsequent figure displays the flexible modelling of continuous variables.

| Variable | HR | 2.5% CI | 97.5% CI | Std. error | P value |
| --- | --- | --- | --- | --- | --- |
| LowC | 2.084 | 0.989 | 4.392 | 0.380 | 0.054 |
| HighC | 1.045 | 0.521 | 2.098 | 0.355 | 0.900 |
| Male | 0.986 | 0.664 | 1.463 | 0.201 | 0.943 |
| Age | 1.013 | 0.997 | 1.029 | 0.008 | 0.117 |
| Invasiv | 0.414 | 0.146 | 1.176 | 0.533 | 0.098 |
| SOFA | 1.180 | 1.130 | 1.232 | 0.022 | 0.000 |
| Creatinine | 0.816 | 0.661 | 1.008 | 0.108 | 0.060 |

**Supp. Table 4: Results of Cox proportional hazards model with confounders,** reference group: NoC, no corticosteroid dose; HR, hazard ratio; Cl, confidence limit; Std. Error, standard error; LowC, low corticosteroid dose; HighC, high corticosteroid dose


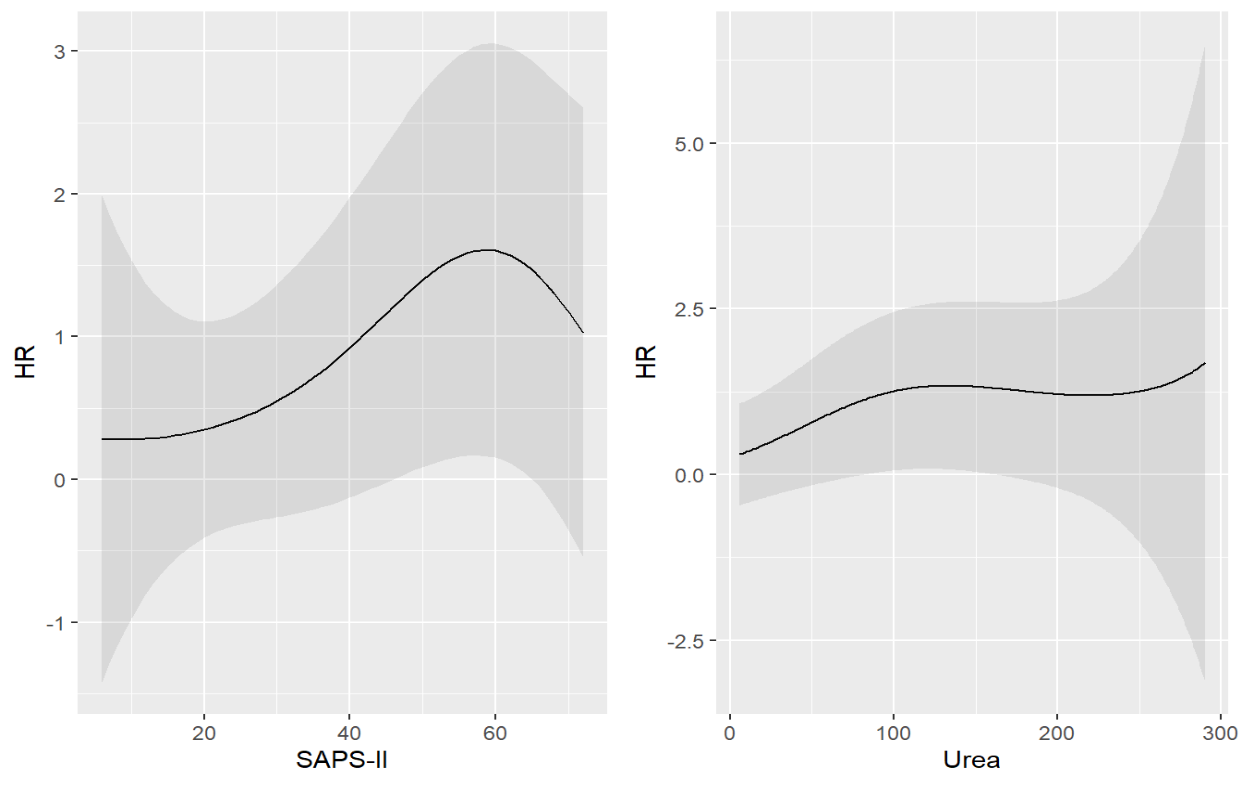


**Supp. Figure 4: Confounders that were modelled with b-splines and 3 degrees of freedom.**

5a) 5b)


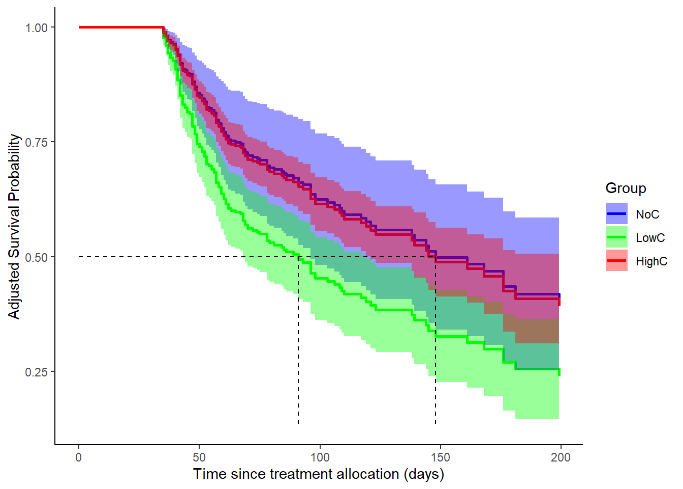

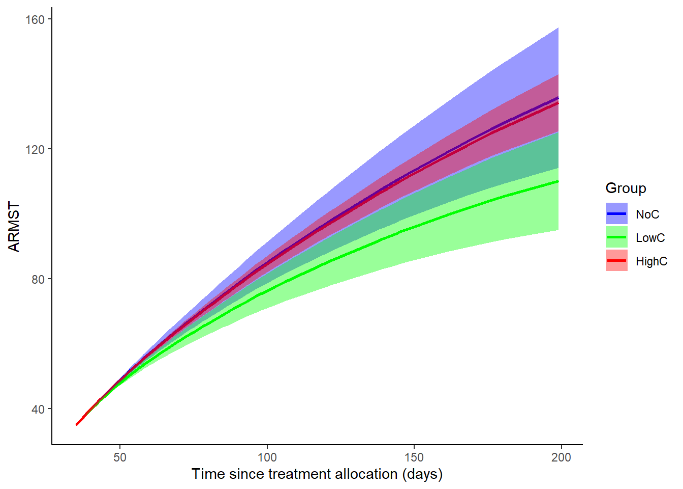


**Supp. Figure 5: Overall survival.** Adjusted survival curves (5a) for the landmarking time point of 35 days showing the overall survival of COVID-19 patients treated with either NoC (blue line), LowC (green line) or HighC (red line); n= 486. **5a.** Adjusted Survival Probability and **5b.** Adjusted restricted mean survival time. NoC, no corticosteroid dose; LowC, low corticosteroid dose; HighC, high corticosteroid dose; ARMST, adjusted restricted mean survival time.

**
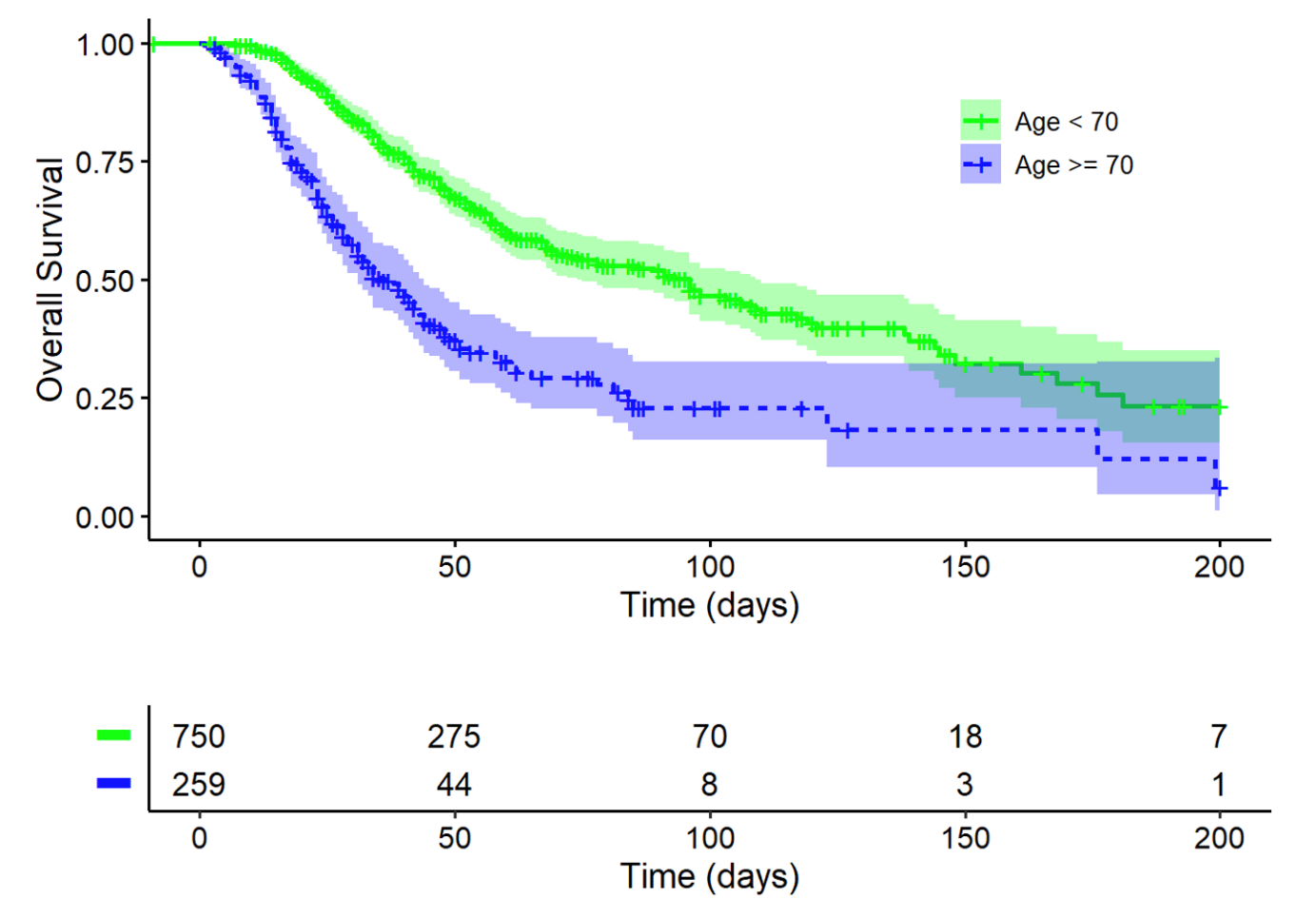
**

**Supp. Figure 6: Overall survival for all three cohorts without adjustment for patients under and over 70 years** Patients under 70 years are represented by the green line, and patients aged 70 years or older are represented by the blue line.

1. Westreich D, Greenland S: **The table 2 fallacy: presenting and interpreting confounder and modifier coefficients**. *Am J Epidemiol* 2013, **177**(4):292-298.

2. Harrell FE: **Regression modeling strategies. With applications to linear models, logistic regression, and survival analysis.**
